# Contemporary practice patterns in IDH-mutant glioma management: a multidisciplinary multi-institutional survey

**DOI:** 10.1007/s11060-026-05630-3

**Published:** 2026-06-08

**Authors:** Tyler A. Lanman, L. Nicolas Gonzalez Castro, Haley K. Perlow, Gilbert Youssef, Roy E. Strowd, Michael T. Milano, Marina Kushnirsky, Ankush Bhatia, Patrick Y. Wen, Julie J. Miller, Benjamin J. Rich

**Affiliations:** 1 Department of Neurology, University of Washington, 1959 NE Pacific St., Seattle, WA 98195 USA; 2 Center for Tumors of the Nervous System, Mass General Brigham Cancer Institute, Boston, MA USA; 3 Department of Neurology, Harvard Medical School, Boston, MA USA; 4 Center for Neuro-Oncology, Dana-Farber Cancer Institute, Boston, MA USA; 5 Department of Radiation Oncology, University Hospitals Seidman Cancer Center, Case Western Reserve University, Cleveland, OH USA; 6 Department of Neurology and Cancer Medicine, Wake Forest University School of Medicine, Winston Salem, NC USA; 7 Department of Radiation Oncology, University of Rochester Medical Center, Rochester, NY USA; 8 Department of Neurology, University of Miami, Miami, FL USA; 9 Department of Neurology, University of Wisconsin School of Medicine and Public Health, Madison, WI USA; 10 Department of Radiation Oncology, University of Miami/Sylvester Comprehensive Cancer Center, Miami, FL USA

**Keywords:** IDH-mutant glioma, Astrocytoma, Oligodendroglioma, Glioma, IDH, IDH inhibitor, Isocitrate dehydrogenase, Survey, Practice patterns

## Abstract

**Purpose:**

IDH-mutant glioma management has been rapidly evolving following the 2024 FDA approval of the mutant IDH inhibitor (IDHi), vorasidenib. However, optimal treatment decisions for many specific scenarios remain unclear. The aim of this study was to provide insight into how clinicians collectively approach different clinical scenarios and evaluate how demographic and professional backgrounds influence decision-making for IDH-mutant glioma.

**Methods:**

A survey was developed by a team of neuro-oncologists and radiation oncologists and distributed via email and X (formerly Twitter) to clinicians who treat patients with IDH-mutant glioma (neuro/medical oncologists, radiation oncologists, and neurosurgeons). The survey included demographic questions and 10–13 case-based clinical scenarios with standardized response options. We compared responses between neuro/medical oncologists and radiation oncologists and performed univariable regression to identify predictors of treatment preference and IDHi familiarity/enthusiasm.

**Results:**

A total of 153 clinicians (58% neuro/medical-oncologists, 34% radiation oncologists, 8% neurosurgeons) completed the survey. Five of ten scenarios reached consensus (> 75% agreement on a treatment option), while the remainder demonstrated heterogeneity. Compared to neuro/medical oncologists, radiation oncologists were less likely to recommend IDHi (IRR 0.57, *p* < 0.001) and more likely to recommend radiotherapy (IRR 1.54, *p* < 0.001) or chemoradiotherapy (IRR 1.47, *p* < 0.001). Neuro-oncologists and neurosurgeons reported the most and least familiarity with IDHi, respectively, whereas medical oncologists and radiation oncologists reported the most and least enthusiasm for IDHi, respectively.

**Conclusions:**

Substantial variation exists in real-world management of IDH-mutant glioma across specialties and institutions. While several clinical scenarios demonstrated strong consensus, others revealed uncertainty, underscoring the need for ongoing multidisciplinary collaboration and further advances in evidence-based consensus to guide clinical decisions.

**Supplementary Information:**

The online version contains supplementary material available at 10.1007/s11060-026-05630-3.

## Introduction

Isocitrate dehydrogenase (IDH)-mutant gliomas represent a unique subset of brain tumors (astrocytoma and oligodendroglioma) defined by the presence of a pathogenic IDH mutation [1]. These tumors are often diagnosed in the third to fourth decade of life and, although associated with a more favorable prognosis than IDH-wild type glioblastoma, they remain incurable [1]. Treatment has traditionally involved a combination of surgery (aiming for maximal safe resection), radiotherapy (RT), and chemotherapy (often using temozolomide [TMZ] or a regimen of procarbazine, lomustine and vincristine [PCV]) [1]. In some cases, a “watch-and-wait” approach following surgery has been considered appropriate, acknowledging that both chemotherapy and radiotherapy can be associated with both short- and long-term toxicity [2–4]. However, the clinical landscape for IDH-mutant glioma has evolved rapidly in recent years. The 2021 WHO Classification of Tumors of the Central Nervous System (WHO CNS5) introduced new molecularly-based diagnostic criteria for these tumors [5]. Ongoing trials (e.g. CODEL, NRG-BN005, etc.) are poised to reshape clinical practice but these results are still pending. The Phase 3 INDIGO trial reported significantly improved progression free survival (PFS) and time to next intervention in select patients with IDH-mutant glioma (measurable non-enhancing disease who were felt not to need immediate treatment with radiation and/or chemotherapy) treated with the mutant IDH inhibitor (IDHi) vorasidenib, compared to placebo [6]. These results led to FDA approval of vorasidenib in 2024 for the treatment of CNS WHO grade 2 IDH-mutant glioma, representing a paradigm shift in the management of these tumors [7]. Despite these initially promising developments, many key clinical questions remain unanswered. For instance, overall survival (OS) data from INDIGO are not yet mature and will likely be confounded by high crossover rates [8]. Additionally, vorasidenib was not compared against chemoradiotherapy or chemoradiotherapy plus vorasidenib. Furthermore, many specific clinical scenarios fall outside the INDIGO trial’s inclusion criteria, making extrapolation of its findings unclear [8].

In this context, real-world decision-making often relies on multidisciplinary management, clinical judgement, institutional culture, and interpretation of limited available data. We hypothesized that this has resulted in considerable practice variation across specialties, institutions, and regions. To better understand contemporary treatment preferences for IDH-mutant glioma, we conducted a survey of physicians in the fields of neuro/medical-oncology, radiation oncology, and neurosurgery. The survey presented case-based clinical scenarios to assess how clinicians weigh factors such as age, tumor grade, and extent of resection in their treatment recommendations. Our aim was to provide insight into current clinical thinking, highlight areas of consensus versus uncertainty, and explore how demographic and professional backgrounds influence decision-making in the field of IDH-mutant glioma.

## Methods

A *de novo* survey instrument was created by a multi-institutional team of neuro-oncologists and radiation oncologists. It was implemented with Google Forms and was distributed via Society for Neuro-Oncology’s social media (X; formerly known as Twitter) and direct email outreach to brain tumor specialists at all 73 National Cancer Institute (NCI)-designated cancer centers in the United States. Survey participants were academic and community physicians (neuro-oncologists, medical oncologists, radiation oncologists, and neurosurgeons) familiar with the treatment of IDH-mutant gliomas. The survey was open from April 29, 2025, to August 1, 2025. The authors of this manuscript did not participate in the survey to avoid bias.

The survey consisted of the following (Tables 1, 2, 3 and 4):

1. Demographic and clinical practice questions.

2. Ten case-based clinical scenarios (plus 1–3 extra questions depending on specialty).

3. Additional questions regarding IDHi use, familiarity, and enthusiasm.

**Table 1:** Demographics and professional characteristics of survey participants

Characteristic	Overall *N* = 153
**Specialty**	
Neuro-Oncologist	81 (53%)
Radiation Oncologist	52 (34%)
Neurosurgeon	13 (8.5%)
Medical Oncologist	7 (4.6%)
**Sex**	
Female	55 (36%)
Male	93 (61%)
Other/decline to state	5 (3.3%)
**Years Practicing**	
<5 years	44 (29%)
5–10 years	48 (31%)
10–20 years	37 (24%)
>20 years	24 (16%)
**New patients per month**	
1–2 patients	77 (50%)
3–4 patients	34 (22%)
4 + patients	42 (27%)
**US Region**	
West	33 (22%)
Midwest	33 (22%)
Northeast	56 (37%)
South	24 (16%)
I practice outside the US	7 (4.6%)
**International Region**	
Asia	1 (0.7%)
Europe	3 (2.0%)
North America	148 (97%)
South/Central America	1 (0.7%)
**Practice Setting**	
Academic medical institution	132 (86%)
Non-academic hospital	3 (2.0%)
Free-standing non-hospital	2 (1.3%)
Hybrid model	16 (10%)
**Community Setting**	
Urban	121 (79%)
Suburban	16 (10%)
Rural	2 (1.3%)
Mixed locales	14 (9.2%)
**Tumor Board Frequency per Month**	
Never	1 (0.7%)
< Once	6 (3.9%)
Once	13 (8.5%)
> Once	133 (87%)

**Table 2:**
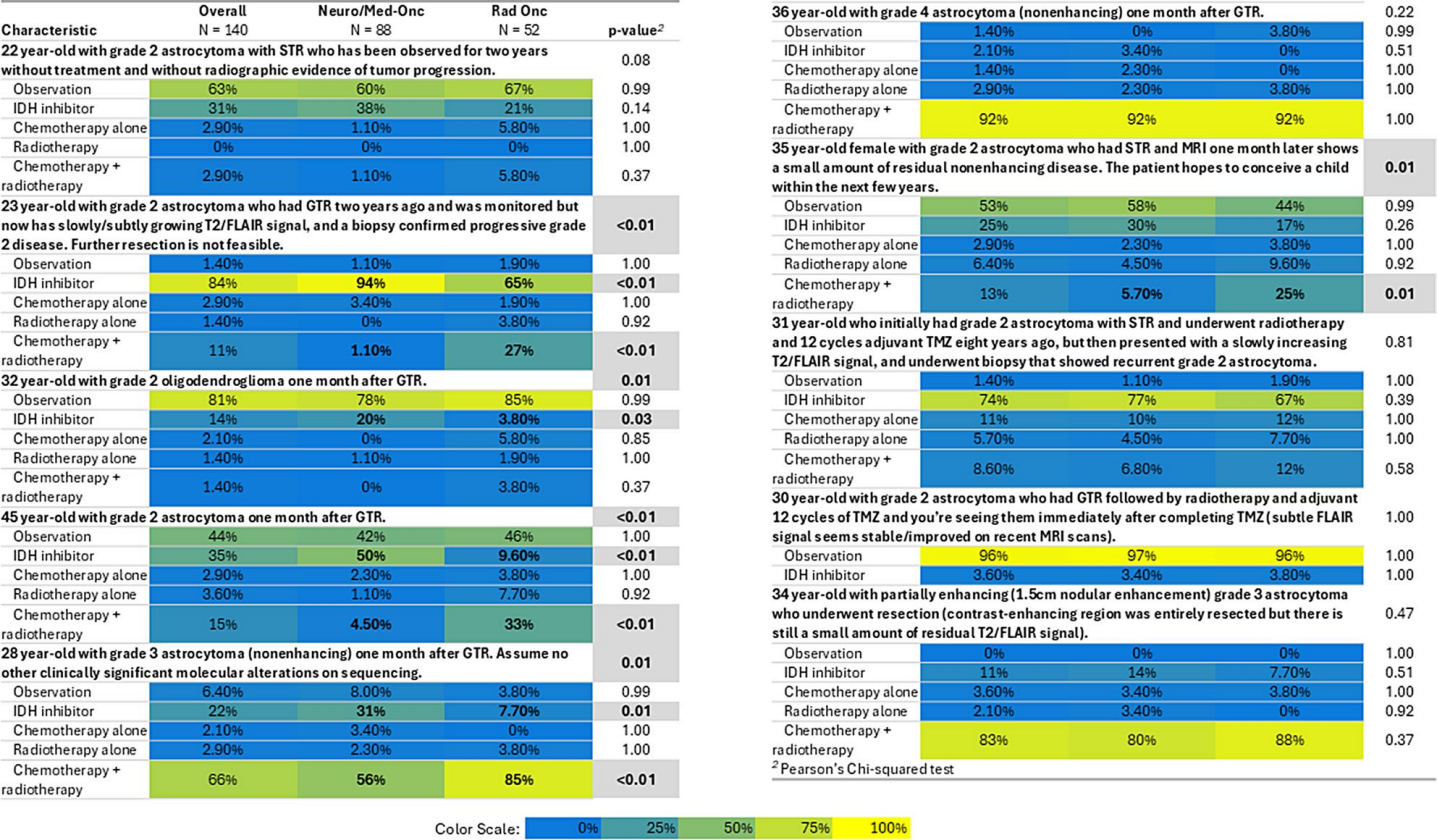
Comparison of responses between neuro/medical-oncologists and radiation oncologists. A color scale illustrates popularity of each response for each question, ranging from blue (not popular) to yellow (most popular). Significant differences between neuro/medical oncologists and radiation oncologists on both questions and individual answers are highlighted in gray. GTR = gross total resection, IDH = isocitrate dehydrogenase, STR = subtotal resection, TMZ = temozolomide

Possible management options for most clinical scenarios were: “observation,” “IDH inhibitor,” “chemotherapy alone,” “radiotherapy alone,” or “chemotherapy + radiotherapy.” Regarding Scenario 6, for neuro/medical oncologists, the “chemotherapy + radiotherapy” option was provided as the following different options: “Radiotherapy followed by 6 cycles TMZ, “Radiotherapy followed by 12 cycles TMZ,” “Radiotherapy with concurrent and 6 cycles adjuvant TMZ,” and “Radiotherapy with concurrent and 12 cycles adjuvant TMZ.” Regarding Scenario 7, for the neuro/medical-oncologists, the “chemotherapy alone” option was provided as the following different options: “TMZ alone,” “CCNU alone,” and “PCV alone.” For Scenario 9, the only possible responses were “Observation” and “IDH inhibitor” since the patient had already just received chemotherapy and radiotherapy. Scenarios 11–14 were only answered by radiation oncologists as they pertained to radiation volume delineation and dosing (Table 3). Scenarios 15–16 were only answered by neuro/medical-oncologists (Table 4). Neurosurgeons completed the general case-based clinical scenarios but were not asked specialty-specific questions related to detailed chemotherapy selection or radiation planning. To measure clinical management differences in an exploratory analysis, responses to scenarios were assigned a clinical aggressiveness score that was aggregated for each participant. The clinical aggressiveness score was defined as: “observation” = 0 points, “IDH inhibitor” = 1 point, “chemotherapy alone” = 2 points, “radiotherapy only” = 2 points, “chemoradiotherapy” = 3 points.

**Table 3:** Results from questions specific to radiation oncologists

Characteristic	Overall *N* = 52	Not Academic *N* = 7	Academic *N* = 45
**(11) What dose of radiotherapy do you give patients with WHO grade 2 oligodendroglioma?**			
45–49 Gy	2 (3.8%)	2 (29%)	0 (0%)
49.1–53 Gy	14 (27%)	3 (43%)	11 (24%)
53.1–57 Gy	36 (69%)	2 (29%)	34 (76%)
57.1–61 Gy	0 (0%)	0 (0%)	0 (0%)
**(12) What dose of radiotherapy do you give patients with WHO grade 2 astrocytoma?**			
45–49 Gy	2 (3.8%)	2 (29%)	0 (0%)
49.1–53 Gy	9 (17%)	2 (29%)	7 (16%)
53.1–57 Gy	37 (71%)	2 (29%)	35 (78%)
57.1–61 Gy	4 (7.7%)	1 (14%)	3 (6.7%)
**(13) What is your clinical target volume (CTV) margin around FLAIR hyperintensity for WHO grade 2 oligodendroglioma?**			
0 cm	1 (1.9%)	0 (0%)	1 (2.2%)
0.5 cm	7 (13%)	1 (14%)	6 (13%)
1 cm	34 (65%)	3 (43%)	31 (69%)
1.5 cm	9 (17%)	2 (29%)	7 (16%)
2 cm	1 (1.9%)	1 (14%)	0 (0%)
**(14) What is your clinical target volume (CTV) margin around FLAIR hyperintensity for WHO grade 2 astrocytoma?**			
0 cm	1 (1.9%)	0 (0%)	1 (2.2%)
0.5 cm	7 (13%)	2 (29%)	5 (11%)
1 cm	36 (69%)	4 (57%)	32 (71%)
1.5 cm	8 (15%)	1 (14%)	7 (16%)
2 cm	0 (0%)	0 (0%)	0 (0%)

Responses are shown based on academic vs non-academic clinical setting and overall.

**Table 4:** Results from questions specific to neuro/medical-oncologists

Characteristic	Overall *N* = 88	Not Academic *N* = 13	Academic *N* = 75
**(6) 36 year-old with grade 4 astrocytoma (nonenhancing) one month after GTR.**			
IDH inhibitor	3 (3.4%)	1 (7.7%)	2 (2.7%)
Chemotherapy alone	2 (2.3%)	1 (7.7%)	1 (1.3%)
Radiotherapy alone	2 (2.3%)	2 (15%)	0 (0%)
Radiotherapy followed by 6 cycles TMZ	6 (6.8%)	0 (0%)	6 (8.0%)
Radiotherapy followed by 12 cycles TMZ	16 (18%)	3 (23%)	13 (17%)
Radiotherapy with concurrent and 6 cycles adjuvant TMZ	26 (30%)	4 (31%)	22 (29%)
Radiotherapy with concurrent and 12 cycles adjuvant TMZ	33 (38%)	2 (15%)	31 (41%)
**(7) 35 year-old female with grade 2 astrocytoma who had STR and MRI one month later shows a small amount of residual nonenhancing disease. The patient hopes to conceive a child within the next few years.**			
Observation	51 (58%)	4 (31%)	47 (63%)
IDH inhibitor	26 (30%)	6 (46%)	20 (27%)
TMZ alone	0 (0%)	0 (0%)	0 (0%)
CCNU alone	1 (1.1%)	1 (7.7%)	0 (0%)
PCV alone	1 (1.1%)	1 (7.7%)	0 (0%)
Radiotherapy alone	4 (4.5%)	0 (0%)	4 (5.3%)
Chemotherapy + radiotherapy	5 (5.7%)	1 (7.7%)	4 (5.3%)
**(15) 29 year-old with grade 2 oligodendroglioma who underwent GTR and was subsequently started on an IDH inhibitor but now has small amount of FLAIR signal around the resection cavity after six months that is more prominent on the current scan compared to the past two scans.**			
Continue IDH inhibitor for another 1–2 scans and reassess	56 (64%)	5 (38%)	51 (68%)
Stop IDH inhibitor and start chemotherapy	2 (2.3%)	1 (7.7%)	1 (1.3%)
Stop IDH inhibitor and start radiotherapy	2 (2.3%)	1 (7.7%)	1 (1.3%)
Stop IDH inhibitor and start radiotherapy and chemotherapy	6 (6.8%)	1 (7.7%)	5 (6.7%)
Ask surgeons for evaluation of resection/biopsy	22 (25%)	5 (38%)	17 (23%)
**(16) Since the approval of vorasidenib, for patients who had been stable taking ivosidenib**,** have you generally been switching them to vorasidenib?**			
Yes	21 (24%)	5 (38%)	16 (21%)
No	20 (23%)	2 (15%)	18 (24%)
Case-by-case basis	32 (36%)	5 (38%)	27 (36%)
I did not have patients taking ivosidenib	15 (17%)	1 (7.7%)	14 (19%)

Responses are shown based on academic vs non-academic clinical setting and overall. CCNU = lomustine, GTR = gross total resection, IDH = isocitrate dehydrogenase, PCV = procarbazine, lomustine and vincristine, STR = subtotal resection, TMZ = temozolomide

Pearson’s chi-squared test was used to compare responses between specialties. The Benjamini-Hochberg method was used to control for false discovery rate (FDR) where applicable. Univariable Poisson regression was used to evaluate predictors of specific response counts, and univariable linear regression was used to assess predictors of IDHi familiarity and enthusiasm (on a scale of 1 to 5). A p-value < 0.05 was considered statistically significant. Analyses were performed using R (version 4.2.2).

## Results

A total of 153 clinicians completed the survey. Respondent demographics and clinical practice characteristics are detailed in Table 1. Most were neuro-oncologists (53%) or radiation oncologists (34%), identified as male (61%), practiced at an academic medical institution (86%) and/or in an urban setting (79%). Respondents were geographically diverse within the United States, and 4.6% practiced outside the US.

**Fig. 1:**
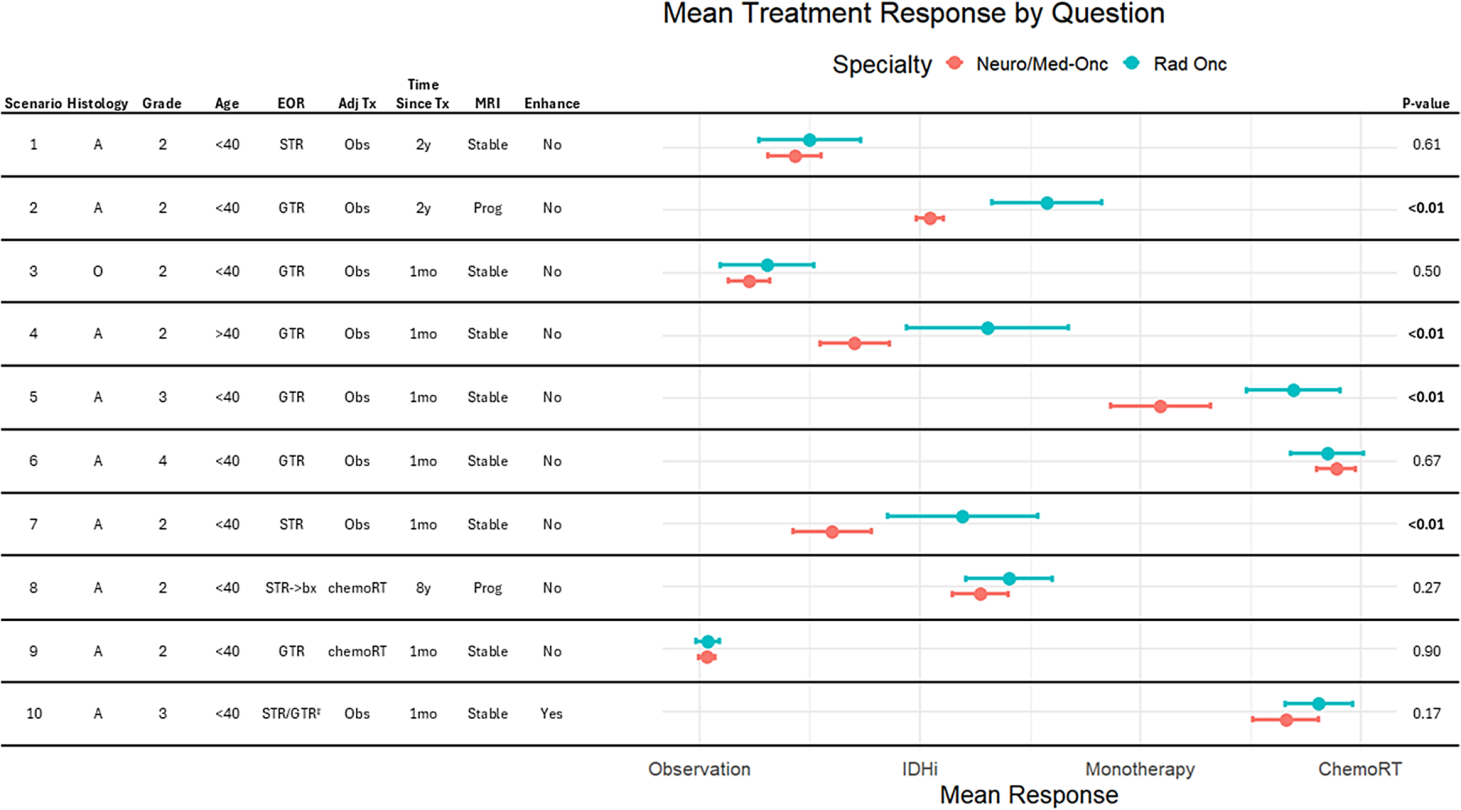
Clinical aggressiveness for each question based on specialty. Clinical factors that distinguish each question are provided in the table on the left. The mean response on a scale ranging from observation to chemoradiotherapy are visualized for each question for both neuro-medical oncologists and radiation oncologists. Error bars represent 95% confidence intervals. P-values are computed with two sample t-test. #STR of T2 signal and GTR of enhancing signal. A = astrocytoma, Adj = adjuvant, bx = biopsy, chemoRT = chemoradiotherapy. EOR = extent of resection, GTR = gross total resection, O = oligodendroglioma, Obs = observation, prog = progression, STR =subtotal resection, Tx = treatment

Aggregate recommendations for each clinical scenario, based on specialty and practice setting, are shown in Supplementary Tables 1 and 2. As a major aim of this project was to compare responses from neuro-oncologists/medical-oncologists to those of radiation oncologists, Table 2; Fig. 1 were created to highlight similarities and differences in responses. Five of ten scenarios (scenarios 2, 3, 6, 9, and 10) demonstrated strong overall consensus (defined here as > 75% for a specific response), while the remaining scenarios (scenarios 1, 4, 5, 7, and 8) revealed practice heterogeneity (≤ 75% for a specific response). In scenarios 2, 3, 4, 5, and 7, responses from neuro/medical-oncologists significantly differed from those of radiation oncologists. In scenario 2 in particular, 94% of neuro/med-oncologists agreed on IDH inhibitor whereas radiation oncologists were more split with only 65% recommending IDH inhibitor and 27% recommending chemotherapy and radiotherapy.

**Fig. 2:**
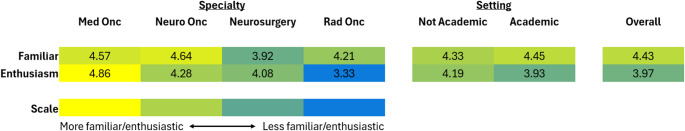
Average responses for IDHi Familiarity and Enthusiasm. Survey participants were asked to rate their level of enthusiasm and their level of knowledge regarding IDH (isocitrate dehydrogenase) inhibitors from a scale from 1 to 5. Average responses are shown based on specialty, academic vs nonacademic clinical setting, and overall. A color scale ranges from yellow (more familiar/enthusiastic) to blue (less familiar/enthusiastic)

Neuro-oncologists reported the highest familiarity with using IDHi (average 4.64 on a scale of 1–5) while neurosurgeons reported the least (3.92); the overall average familiarity was 4.43 (Fig. 2). Medical oncologists reported the highest enthusiasm for IDHi (4.86) whereas radiation oncologists reported the least enthusiasm (3.33); the overall average enthusiasm was 3.97 (Fig. 2).

Questions answered only by radiation-oncologists are presented in Table 3. Most radiation oncologists recommended a dose of 53.1 to 57 Gy for both grade 2 oligodendroglioma (69%) and astrocytoma (71%). Most recommended utilizing a 1 cm clinical target volume (CTV) margin around fluid-attenuated inversion recovery (FLAIR) signal for both grade 2 oligodendroglioma (65%) and astrocytoma (69%).

Questions answered only by neuro/medical oncologists are shown in Table 4. For a grade 4 nonenhancing astrocytoma after gross total resection (GTR) (scenario 6), chemotherapy-specific responses were split, with 38% recommending RT with concurrent and 12 cycles adjuvant TMZ, 30% recommending RT with concurrent and 6 cycles adjuvant TMZ, and 18% recommending RT followed by 12 cycles adjuvant TMZ. For a patient with grade 2 astrocytoma who wishes to conceive a child (scenario 7), 88% recommended observation or IDHi so it was not possible to identify a recommendation for a specific chemotherapy (e.g. TMZ, CCNU, PCV). For a patient with grade 2 oligodendroglioma with GTR who has been on IDHi but who has a small amount of FLAIR signal (scenario 15), 64% chose to continue the IDHi for another 1–2 scans and reassess whereas 25% opted to ask surgeons for a resection/biopsy; only 11% chose to empirically stop the IDHi and pursue another treatment course. Finally, for the question of IDHi preference since the approval of vorasidenib (scenario 16), responses were mixed, with 24% saying that they empirically have been switching patients from ivosidenib to vorasidenib, 23% said they are continuing with ivosidenib in patients whose glioma is stable, and 36% are making case-by-case decisions.

Exploratory univariable regression was performed to evaluate associations between provider demographics and treatment preferences. Academic clinicians were more likely to recommend observation across the presented clinical scenarios (Supplementary Table 3). Radiation oncologists and those in urban settings were less likely to recommend IDHi, while greater IDHi familiarity and enthusiasm were associated with higher likelihood to make this recommendation (Supplementary Table 4). Radiation oncologists, physicians in urban settings, and those with lower IDHi enthusiasm were more likely to recommend radiotherapy (Supplementary Table 5). Academic clinicians, radiation oncologists, urban clinicians, those who attend tumor board meetings more frequently and those with lower IDHi enthusiasm were more likely to recommend chemoradiotherapy (Supplementary Table 6).

On a scale of “clinical aggressiveness” (ranging from observation to chemoradiotherapy), radiation oncologists were more aggressive and higher IDHi enthusiasm was associated with lower clinical aggressiveness (Supplementary Table 7). Being a radiation oncologist or neurosurgeon and practicing outside the US were associated with less familiarity with IDHi whereas higher caseload of patients with IDH-mutant glioma was associated with more familiarity (Supplementary Table 8). Being a radiation oncologist and practicing in the South of the United States were associated with lower enthusiasm for IDHi (Supplementary Table 9).

## Discussion

The treatment of IDH-mutant glioma has been rapidly evolving. While the INDIGO phase 3 trial demonstrated a PFS benefit with vorasidenib compared to placebo, strict inclusion criteria from this study limits its external validity. Real-world IDHi studies have been published but their results are inherently limited by their retrospective design and associated selection biases [9–11]. On the other hand, the RTOG 9802 trial has over 10 years of follow-up and showed an overall survival benefit with the addition of PCV to radiation therapy in patients with high-risk IDH-mutant gliomas regardless of 1p19q codeletion status [12,13]. IDH-mutant glioma management guidelines published in the wake of the INDIGO trial reflect the differences and uncertainty in approaching this entity [1, 14–17]. Clinicians often must decide between different treatment recommendations in specific clinical situations not adequately addressed by the literature, balancing risks of potential neurotoxicity with uncertainty regarding long-term outcomes for vorasidenib and how sequencing of treatments influences survival. Our survey aimed to clarify practice patterns across specialties and institutions.

In our survey, half of the clinical scenarios reached overall consensus, providing some general guidance for those facing such situations. For instance, the strongest consensus to start an IDHi was for a patient with grade 2 astrocytoma with biopsy-proven recurrent grade 2 astrocytoma with slowly increasing FLAIR signal (scenario 2). As this scenario closely matches the INDIGO trial population, the consensus suggests that clinicians are generally comfortable extending those results to comparable real-world cases. In a 32 year-old patient with grade 2 oligodendroglioma after a GTR (scenario 3), the consensus was to observe. Patients with low-grade IDH-mutant 1p/19/q codeletion gliomas and no high-risk factors enjoyed a median progression-free survival of 8.3 years with observation on RTOG 9802 [18]. Thus, although IDHi are well-tolerated, starting an IDHi in this setting carries a risk of potential side effects from long term medication use for a tumor that might not recur for many years. The consensus for a patient with grade 4 IDH-mutant astrocytoma (scenario 6) was chemotherapy and radiation. This is acknowledged as an entity distinct from glioblastoma since 2021 and an optimal treatment approach has not been explicitly examined in clinical trials. For instance, should it be managed like a lower grade IDH-mutant glioma with a long course of adjuvant chemotherapy or like a glioblastoma with both concurrent and adjuvant chemotherapy [19, 20] For this scenario (scenario 6), neuro/medical-oncologists were divided on how to structure therapy although most recommended chemoradiotherapy followed by 12 cycles of adjuvant temozolomide. For a patient with grade 2 astrocytoma who just completed chemoradiotherapy (scenario 9), the consensus was to observe. Currently, it remains unclear whether or not IDH inhibition after radiotherapy and chemotherapy improves outcomes but two trials are actively investigating this topic (NCT06809322, 1/22/25; NCT05303519, 3/13/22). For a patient with a grade 3 astrocytoma and enhancing disease that had been resected (scenario 10), the consensus was to recommend chemoradiotherapy. The CATNON trial demonstrated an overall survival benefit by adding adjuvant chemotherapy (temozolomide) following post-operative radiation therapy in patients with IDH-mutant 1p/19q non-co-deleted high-grade gliomas [21]. This is also in accordance with multiple lines of evidence suggesting that ivosidenib and vorasidenib do not work well in patients with enhancing disease [22, 23] although it is unclear whether a small degree of enhancement that is completely resected would convey the same degree of resistance to IDHi [10,16]. Furthermore, there is preliminary/emerging evidence that enhancing IDH-mutant glioma tumors might still respond to newer IDHi (i.e. safusidenib, olutasidenib) [24,25].

Conversely, the other half of clinical scenarios lacked consensus, highlighting areas for future research. Although the case with the grade 2 astrocytoma with a subtotal resection (STR) but stable disease for 2 years (scenario 1) technically would have fit the inclusion criteria for INDIGO, the majority favored observation over IDHi (likely due to the long radiographic stability in this case) although there was not a strong consensus. There was not a consensus for a 45 year-old patient with grade 2 astrocytoma after GTR (scenario 4) (observation was only slightly favored over IDHi), which contrasts with the parallel scenario with the 32 year-old patient with grade 2 oligodendroglioma (scenario 3) where consensus was to favor observation. This discrepancy could reflect differences in how we treat astrocytoma vs. oligodendroglioma or could also reflect the historical cutoff of 40 years old for determining “high-risk” vs. “low-risk.” However, some argue that such an age cutoff, defined as high-risk by RTOG 9802, should not be used in the modern era, and National Comprehensive Cancer Network (NCCN) guidelines have now removed this cutoff from their risk stratification schema [16]. For a patient with nonenhancing grade 3 astrocytoma after GTR, the non-consensus majority chose to give chemoradiotherapy although a sizeable minority were comfortable offering an IDHi (scenario 5). This is a controversial topic because although the INDIGO trial only included patients with grade 2 tumors (reflected by the current FDA indications for grade 2 tumors), some providers feel that extrapolation to grade 3 tumors could be warranted in a case-by-case basis given that in the modern molecular era, there might not be as much of a distinction between grade 2 and 3 tumors as previously thought [16, 24, 26]. A recent retrospective analysis did not find significant differences in responses to ivosidenib between grade 2 and 3 IDH-mutant glioma [10]. Accordingly, August 2025 NCCN guidelines include IDH inhibitors as a potential option for select patients with grade 3 oligodendroglioma or astrocytoma with the caveat that this is category 2B evidence. A clinical trial is currently evaluating the safety of vorasidenib with temozolomide after chemoradiation in high-grade gliomas (NCT 06478212, 6/17/2024). Finally, in the scenario where a patient with a grade 2 astrocytoma had STR but hopes to conceive a child (scenario 7), observation was the non-consensus majority although a sizeable minority recommended an IDHi. In this setting, there is a suggestion that IDHi might detrimentally impact fertility and potentially cause birth defects, although this is limited to unpublished preclinical data where rodents were exposed to IDHi at much higher concentrations than the equivalent human dose [27, 28]. Regardless, the FDA recommends that patients do not get pregnant while taking IDHi and discontinue the IDHi for a period of time before attempting to conceive [27]. Further, many physicians recommend fertility preservation measures prior to IDH inhibition, much like what is done in anticipation of chemotherapy.

The survey results also highlight a divergence between treatment recommendations from different specialties. Notably, radiation oncologists were significantly less likely than neuro/medical oncologists to recommend IDHi and more likely to recommend radiotherapy with or without chemotherapy. This suggests that neuro/medical-oncologists are more willing to introduce IDHi into clinical practice than their radiation oncology counterparts, a fact also reflected in the discrepancy of averages scores for enthusiasm for IDHi. This mirrors differences in IDHi enthusiasm seen in society guidelines [1, 14, 15]. Other demographic factors of respondents were not as impactful for differentiating responses. For instance, other than IDHi enthusiasm, US region did not significantly impact responses, and the longitudinal experience of the provider did not significantly impact any responses.

This study has several limitations. First, the survey reflects stated treatment preferences rather than real‑world clinical behavior, which is influenced by factors not fully captured in survey scenarios. In addition, the treatment preferences reported may not remain representative of future practice patterns given maturing data in this realm. Second, selection bias is inherent to the survey methodology, acknowledging that the participant pool was skewed toward academic centers and clinicians familiar with IDH inhibitor use. Third, the survey instrument was developed *de novo* by a multidisciplinary team, and not formally validated. Finally, the clinical aggressiveness score and consensus threshold (> 75% agreement) were exploratory, intended to facilitate comparisons rather than imply treatment appropriateness or deviation from standard of care.

Nonetheless, this study provides a timely snapshot of multidisciplinary practice variation during a period of rapid change in IDH‑mutant glioma management, and highlights areas of consensus and uncertainty that warrant further investigation. As it is unlikely that formal trials will be able to answer several important nuanced clinical questions regarding IDH-mutant glioma, it will be important to create and analyze carefully constructed patient registries to guide optimal practice. Finally, we emphasize that multidisciplinary collaboration is essential for meaningful progress in this field, especially given the different perspectives on treatment held by different specialties. We hope that this work contributes to ongoing dialogue and collaboration across disciplines as the field continues to evolve.

## Supplementary Information

Below is the link to the electronic supplementary material.

Supplementary Material 1

Supplementary Material 2

Supplementary Material 3

Supplementary Material 4

Supplementary Material 5

Supplementary Material 6

Supplementary Material 7

Supplementary Material 8

Supplementary Material 9

Supplementary Material 10

## Data Availability

The datasets generated during and/or analyzed during the current study are available from the corresponding author on reasonable request.
