# Supplementary material for: Contemporary practice patterns in IDH-mutant glioma management: a multidisciplinary multi-institutional survey
Source: J Neurooncol. 2026 Jun 8;178(2):54. doi: 10.1007/s11060-026-05630-3 (PMC13246546; doi:10.1007/s11060-026-05630-3)
Supplement: Supplementary file 2 — Supplementary Material 2 [file 11060_2026_5630_MOESM2_ESM.docx]

Supplementary Table 2: Responses based on institution type (academic vs not academic).

| **Characteristic** | **Overall** N = 153 | **Not Academic** N = 21 | **Academic** N = 132 |
| --- | --- | --- | --- |
| **(1) 22 year-old with grade 2 astrocytoma with STR who has been observed for two years without treatment and without radiographic evidence of tumor progression.** |  |  |  |
| Observation | 97 (63%) | 9 (43%) | 88 (67%) |
| IDH inhibitor | 46 (30%) | 7 (33%) | 39 (30%) |
| Chemotherapy alone | 5 (3.3%) | 3 (14%) | 2 (1.5%) |
| Radiotherapy alone | 1 (0.7%) | 1 (4.8%) | 0 (0%) |
| Chemotherapy + radiotherapy | 4 (2.6%) | 1 (4.8%) | 3 (2.3%) |
| **(2) 23 year-old with grade 2 astrocytoma who had GTR two years ago and was monitored but now has slowly/subtly growing T2/FLAIR signal, and a biopsy confirmed progressive grade 2 disease. Further resection is not feasible.** |  |  |  |
| Observation | 3 (2.0%) | 1 (4.8%) | 2 (1.5%) |
| IDH inhibitor | 128 (84%) | 14 (67%) | 114 (86%) |
| Chemotherapy alone | 4 (2.6%) | 3 (14%) | 1 (0.8%) |
| Radiotherapy alone | 2 (1.3%) | 0 (0%) | 2 (1.5%) |
| Chemotherapy + radiotherapy | 16 (10%) | 3 (14%) | 13 (9.8%) |
| **(3) 32 year-old with grade 2 oligodendroglioma one month after GTR.** |  |  |  |
| Observation | 124 (81%) | 12 (57%) | 112 (85%) |
| IDH inhibitor | 22 (14%) | 6 (29%) | 16 (12%) |
| Chemotherapy alone | 3 (2.0%) | 3 (14%) | 0 (0%) |
| Radiotherapy alone | 2 (1.3%) | 0 (0%) | 2 (1.5%) |
| Chemotherapy + radiotherapy | 2 (1.3%) | 0 (0%) | 2 (1.5%) |
| **(4) 45 year-old with grade 2 astrocytoma one month after GTR.** |  |  |  |
| Observation | 67 (44%) | 3 (14%) | 64 (48%) |
| IDH inhibitor | 52 (34%) | 10 (48%) | 42 (32%) |
| Chemotherapy alone | 5 (3.3%) | 3 (14%) | 2 (1.5%) |
| Radiotherapy alone | 6 (3.9%) | 3 (14%) | 3 (2.3%) |
| Chemotherapy + radiotherapy | 23 (15%) | 2 (9.5%) | 21 (16%) |
| **(5) 28 year-old with grade 3 astrocytoma (nonenhancing) one month after GTR. Assume no other clinically significant molecular alterations on sequencing.** |  |  |  |
| Observation | 10 (6.5%) | 2 (9.5%) | 8 (6.1%) |
| IDH inhibitor | 35 (23%) | 5 (24%) | 30 (23%) |
| Chemotherapy alone | 3 (2.0%) | 2 (9.5%) | 1 (0.8%) |
| Radiotherapy alone | 5 (3.3%) | 3 (14%) | 2 (1.5%) |
| Chemotherapy + radiotherapy | 100 (65%) | 9 (43%) | 91 (69%) |
| **(6) 36 year-old with grade 4 astrocytoma (nonenhancing) one month after GTR.** |  |  |  |
| Observation | 2 (1.3%) | 1 (4.8%) | 1 (0.8%) |
| IDH inhibitor | 3 (2.0%) | 1 (4.8%) | 2 (1.5%) |
| Chemotherapy alone | 3 (2.0%) | 1 (4.8%) | 2 (1.5%) |
| Radiotherapy alone | 5 (3.3%) | 5 (24%) | 0 (0%) |
| Chemotherapy + radiotherapy | 140 (92%) | 13 (62%) | 127 (96%) |
| **(7) 35 year-old female with grade 2 astrocytoma who had STR and MRI one month later shows a small amount of residual nonenhancing disease. The patient hopes to conceive a child within the next few years.** |  |  |  |
| Observation | 80 (52%) | 6 (29%) | 74 (56%) |
| IDH inhibitor | 38 (25%) | 7 (33%) | 31 (23%) |
| Chemotherapy alone | 5 (3.3%) | 4 (19%) | 1 (0.8%) |
| Radiotherapy alone | 11 (7.2%) | 0 (0%) | 11 (8.3%) |
| Chemotherapy + radiotherapy | 19 (12%) | 4 (19%) | 15 (11%) |
| **(8) 31 year-old who initially had grade 2 astrocytoma with STR and underwent radiotherapy and 12 cycles adjuvant TMZ eight years ago, but then presented with a slowly increasing T2/FLAIR signal, and underwent biopsy that showed recurrent grade 2 astrocytoma.** |  |  |  |
| Observation | 2 (1.3%) | 1 (4.8%) | 1 (0.8%) |
| IDH inhibitor | 114 (75%) | 10 (48%) | 104 (79%) |
| Chemotherapy alone | 16 (10%) | 3 (14%) | 13 (9.8%) |
| Radiotherapy alone | 8 (5.2%) | 5 (24%) | 3 (2.3%) |
| Chemotherapy + radiotherapy | 13 (8.5%) | 2 (9.5%) | 11 (8.3%) |
| **(9) 30 year-old with grade 2 astrocytoma who had GTR followed by radiotherapy and adjuvant 12 cycles of TMZ and you’re seeing them immediately after completing TMZ (subtle FLAIR signal seems stable/improved on recent MRI scans).** |  |  |  |
| Observation | 148 (97%) | 18 (86%) | 130 (98%) |
| IDH inhibitor | 5 (3.3%) | 3 (14%) | 2 (1.5%) |
| **(10) 34 year-old with partially enhancing (1.5cm nodular enhancement) grade 3 astrocytoma who underwent resection (contrast-enhancing region was entirely resected but there is still a small amount of residual T2/FLAIR signal).** |  |  |  |
| Observation | 2 (1.3%) | 1 (4.8%) | 1 (0.8%) |
| IDH inhibitor | 17 (11%) | 6 (29%) | 11 (8.3%) |
| Chemotherapy alone | 5 (3.3%) | 3 (14%) | 2 (1.5%) |
| Radiotherapy alone | 3 (2.0%) | 2 (9.5%) | 1 (0.8%) |
| Chemotherapy + radiotherapy | 126 (82%) | 9 (43%) | 117 (89%) |
