# Supplementary material for: Contemporary practice patterns in IDH-mutant glioma management: a multidisciplinary multi-institutional survey
Source: J Neurooncol. 2026 Jun 8;178(2):54. doi: 10.1007/s11060-026-05630-3 (PMC13246546; doi:10.1007/s11060-026-05630-3)
Supplement: Supplementary file 3 — Supplementary Material 3 [file 11060_2026_5630_MOESM3_ESM.docx]

Supplementary Table 3: Univariable Poisson regression predicting number of “observation” responses.

| Univariable Poisson regression predicting number of 'Observation' responses | | | | |
| --- | --- | --- | --- | --- |
| **Characteristic** | **N** | **IRR** | **95% CI** | **p-value** |
| **Practice Setting** | 153 |  |  |  |
| Not Academic |  | — | — |  |
| Academic |  | 1.42 | 1.08, 1.90 | **0.015** |
| **Specialty** | 153 |  |  |  |
| Neuro-Oncologist |  | — | — |  |
| Radiation Oncologist |  | 0.99 | 0.82, 1.19 | 0.9 |
| Neurosurgeon |  | 1.06 | 0.78, 1.43 | 0.7 |
| Medical Oncologist |  | 0.69 | 0.40, 1.08 | 0.13 |
| **US Region** | 153 |  |  |  |
| West |  | — | — |  |
| Midwest |  | 0.98 | 0.76, 1.26 | 0.8 |
| Northeast |  | 0.89 | 0.71, 1.12 | 0.3 |
| South |  | 1.01 | 0.77, 1.33 | >0.9 |
| Outside US |  | 0.77 | 0.47, 1.21 | 0.3 |
| **Community Setting** | 153 |  |  |  |
| Not Urban |  | — | — |  |
| Urban |  | 1.14 | 0.92, 1.42 | 0.2 |
| **Years Practicing** | 153 | 1.04 | 0.96, 1.12 | 0.4 |
| **New Patients per Month** | 153 | 1.01 | 0.91, 1.11 | >0.9 |
| **Tumor Board Frequency** | 153 | 1.18 | 0.99, 1.44 | 0.081 |
| **Familiarity with IDH inhibitors** | 153 | 1.00 | 0.89, 1.13 | >0.9 |
| **Enthusiasm about IDH inhibitors** | 153 | 0.98 | 0.90, 1.07 | 0.6 |
