# Supplementary material for: Contemporary practice patterns in IDH-mutant glioma management: a multidisciplinary multi-institutional survey
Source: J Neurooncol. 2026 Jun 8;178(2):54. doi: 10.1007/s11060-026-05630-3 (PMC13246546; doi:10.1007/s11060-026-05630-3)
Supplement: Supplementary file 4 — Supplementary Material 4 [file 11060_2026_5630_MOESM4_ESM.docx]

Supplementary Table 4: Univariable Poisson regression predicting number of “IDH inhibitor” responses.

| Univariable Poisson regression predicting number of 'IDH Inhibitor' responses | | | | |
| --- | --- | --- | --- | --- |
| **Characteristic** | **N** | **IRR** | **95% CI** | **p-value** |
| **Practice Setting** | 153 |  |  |  |
| Not Academic |  | — | — |  |
| Academic |  | 0.90 | 0.70, 1.17 | 0.4 |
| **Specialty** | 153 |  |  |  |
| Neuro-Oncologist |  | — | — |  |
| Radiation Oncologist |  | 0.57 | 0.45, 0.70 | **<0.001** |
| Neurosurgeon |  | 0.79 | 0.55, 1.10 | 0.2 |
| Medical Oncologist |  | 0.99 | 0.64, 1.46 | >0.9 |
| **US Region** | 153 |  |  |  |
| West |  | — | — |  |
| Midwest |  | 0.83 | 0.63, 1.11 | 0.2 |
| Northeast |  | 1.13 | 0.90, 1.44 | 0.3 |
| South |  | 0.72 | 0.52, 1.00 | 0.051 |
| Outside US |  | 0.87 | 0.52, 1.38 | 0.6 |
| **Community Setting** | 153 |  |  |  |
| Not Urban |  | — | — |  |
| Urban |  | 0.77 | 0.62, 0.95 | **0.013** |
| **Years Practicing** | 153 | 1.00 | 0.92, 1.10 | >0.9 |
| **New Patients per Month** | 153 | 1.08 | 0.97, 1.20 | 0.14 |
| **Tumor Board Frequency** | 153 | 0.94 | 0.80, 1.12 | 0.5 |
| **Familiarity with IDH inhibitors** | 153 | 1.19 | 1.04, 1.37 | **0.013** |
| **Enthusiasm about IDH inhibitors** | 153 | 1.37 | 1.23, 1.52 | **<0.001** |
