# Supplementary material for: Contemporary practice patterns in IDH-mutant glioma management: a multidisciplinary multi-institutional survey
Source: J Neurooncol. 2026 Jun 8;178(2):54. doi: 10.1007/s11060-026-05630-3 (PMC13246546; doi:10.1007/s11060-026-05630-3)
Supplement: Supplementary file 5 — Supplementary Material 5 [file 11060_2026_5630_MOESM5_ESM.docx]

Supplementary Table 5: Univariable Poisson regression predicting number of “radiotherapy” responses.

| Univariable Poisson regression predicting number of 'Radiotherapy' responses | | | | |
| --- | --- | --- | --- | --- |
| **Characteristic** | **N** | **IRR** | **95% CI** | **p-value** |
| **Practice Setting** | 153 |  |  |  |
| Not Academic |  | — | — |  |
| Academic |  | 1.09 | 0.84, 1.43 | 0.5 |
| **Specialty** | 153 |  |  |  |
| Neuro-Oncologist |  | — | — |  |
| Radiation Oncologist |  | 1.54 | 1.28, 1.87 | **<0.001** |
| Neurosurgeon |  | 1.13 | 0.79, 1.57 | 0.5 |
| Medical Oncologist |  | 1.02 | 0.62, 1.59 | >0.9 |
| **US Region** | 153 |  |  |  |
| West |  | — | — |  |
| Midwest |  | 1.08 | 0.82, 1.42 | 0.6 |
| Northeast |  | 0.91 | 0.71, 1.17 | 0.5 |
| South |  | 1.21 | 0.91, 1.61 | 0.2 |
| Outside US |  | 1.45 | 0.95, 2.14 | 0.072 |
| **Community Setting** | 153 |  |  |  |
| Not Urban |  | — | — |  |
| Urban |  | 1.30 | 1.03, 1.66 | **0.029** |
| **Years Practicing** | 153 | 0.99 | 0.91, 1.07 | 0.8 |
| **New Patients per Month** | 153 | 0.94 | 0.84, 1.04 | 0.2 |
| **Tumor Board Frequency** | 153 | 1.12 | 0.94, 1.37 | 0.2 |
| **Familiarity with IDH inhibitors** | 153 | 0.89 | 0.80, 1.01 | 0.062 |
| **Enthusiasm about IDH inhibitors** | 153 | 0.81 | 0.74, 0.88 | **<0.001** |
