# Supplementary material for: Contemporary practice patterns in IDH-mutant glioma management: a multidisciplinary multi-institutional survey
Source: J Neurooncol. 2026 Jun 8;178(2):54. doi: 10.1007/s11060-026-05630-3 (PMC13246546; doi:10.1007/s11060-026-05630-3)
Supplement: Supplementary file 6 — Supplementary Material 6 [file 11060_2026_5630_MOESM6_ESM.docx]

Supplementary Table 6: Univariable Poisson regression predicting number of “chemoradiotherapy” responses.

| Univariable Poisson regression predicting number of 'Chemo-radiotherapy responses | | | | |
| --- | --- | --- | --- | --- |
| **Characteristic** | **N** | **IRR** | **95% CI** | **p-value** |
| **Practice Setting** | 153 |  |  |  |
| Not Academic |  | — | — |  |
| Academic |  | 1.48 | 1.09, 2.06 | **0.015** |
| **Specialty** | 153 |  |  |  |
| Neuro-Oncologist |  | — | — |  |
| Radiation Oncologist |  | 1.47 | 1.20, 1.79 | **<0.001** |
| Neurosurgeon |  | 1.00 | 0.68, 1.43 | >0.9 |
| Medical Oncologist |  | 0.68 | 0.36, 1.16 | 0.2 |
| **US Region** | 153 |  |  |  |
| West |  | — | — |  |
| Midwest |  | 1.09 | 0.82, 1.44 | 0.6 |
| Northeast |  | 0.89 | 0.69, 1.16 | 0.4 |
| South |  | 1.17 | 0.87, 1.58 | 0.3 |
| Outside US |  | 1.25 | 0.79, 1.92 | 0.3 |
| **Community Setting** | 153 |  |  |  |
| Not Urban |  | — | — |  |
| Urban |  | 1.39 | 1.08, 1.80 | **0.012** |
| **Years Practicing** | 153 | 1.00 | 0.91, 1.09 | >0.9 |
| **New Patients per Month** | 153 | 0.95 | 0.85, 1.06 | 0.4 |
| **Tumor Board Frequency** | 153 | 1.40 | 1.12, 1.80 | **0.005** |
| **Familiarity with IDH inhibitors** | 153 | 0.97 | 0.86, 1.11 | 0.7 |
| **Enthusiasm about IDH inhibitors** | 153 | 0.79 | 0.73, 0.87 | **<0.001** |
