# Supplementary material for: Contemporary practice patterns in IDH-mutant glioma management: a multidisciplinary multi-institutional survey
Source: J Neurooncol. 2026 Jun 8;178(2):54. doi: 10.1007/s11060-026-05630-3 (PMC13246546; doi:10.1007/s11060-026-05630-3)
Supplement: Supplementary file 7 — Supplementary Material 7 [file 11060_2026_5630_MOESM7_ESM.docx]

Supplementary Table 7: Univariable Poisson regression predicting degree of clinical aggressiveness.

| Univariable Poisson regression predicting clinical aggressiveness | | | | |
| --- | --- | --- | --- | --- |
| **Characteristic** | **N** | **IRR** | **95% CI** | **p-value** |
| **Practice Setting** | 153 |  |  |  |
| Not Academic |  | — | — |  |
| Academic |  | 0.94 | 0.83, 1.07 | 0.3 |
| **Specialty** | 153 |  |  |  |
| Neuro-Oncologist |  | — | — |  |
| Radiation Oncologist |  | 1.24 | 1.13, 1.36 | **<0.001** |
| Neurosurgeon |  | 1.03 | 0.86, 1.21 | 0.8 |
| Medical Oncologist |  | 1.12 | 0.90, 1.38 | 0.3 |
| **US Region** | 153 |  |  |  |
| West |  | — | — |  |
| Midwest |  | 1.08 | 0.94, 1.23 | 0.3 |
| Northeast |  | 1.01 | 0.89, 1.14 | >0.9 |
| South |  | 1.10 | 0.95, 1.27 | 0.2 |
| Outside US |  | 1.23 | 0.99, 1.51 | 0.060 |
| **Community Setting** | 153 |  |  |  |
| Not Urban |  | — | — |  |
| Urban |  | 1.07 | 0.96, 1.20 | 0.2 |
| **Years Practicing** | 153 | 0.98 | 0.94, 1.02 | 0.3 |
| **New Patients per Month** | 153 | 0.97 | 0.92, 1.02 | 0.2 |
| **Tumor Board Frequency** | 153 | 0.99 | 0.92, 1.08 | 0.9 |
| **Familiarity with IDH inhibitors** | 153 | 0.96 | 0.90, 1.02 | 0.2 |
| **Enthusiasm about IDH inhibitors** | 153 | 0.90 | 0.86, 0.94 | **<0.001** |
