# Supplementary material for: Contemporary practice patterns in IDH-mutant glioma management: a multidisciplinary multi-institutional survey
Source: J Neurooncol. 2026 Jun 8;178(2):54. doi: 10.1007/s11060-026-05630-3 (PMC13246546; doi:10.1007/s11060-026-05630-3)
Supplement: Supplementary file 8 — Supplementary Material 8 [file 11060_2026_5630_MOESM8_ESM.docx]

Supplementary Table 8: Univariable linear regression predicting familiarity with IDH inhibitors.

| Univariable Linear regression predicting IDHi Familiarity | | | | |
| --- | --- | --- | --- | --- |
| **Characteristic** | **N** | **Beta** | **95% CI** | **p-value** |
| **Practice Setting** | 153 |  |  |  |
| Not Academic |  | — | — |  |
| Academic |  | 0.11 | -0.22, 0.45 | 0.5 |
| **Specialty** | 153 |  |  |  |
| Neuro-Oncologist |  | — | — |  |
| Radiation Oncologist |  | -0.43 | -0.67, -0.19 | **<0.001** |
| Neurosurgeon |  | -0.72 | -1.1, -0.31 | **<0.001** |
| Medical Oncologist |  | -0.07 | -0.60, 0.46 | 0.8 |
| **US Region** | 153 |  |  |  |
| West |  | — | — |  |
| Midwest |  | 0.03 | -0.31, 0.37 | 0.9 |
| Northeast |  | 0.13 | -0.17, 0.44 | 0.4 |
| South |  | -0.29 | -0.66, 0.08 | 0.13 |
| Outside US |  | -0.74 | -1.3, -0.16 | **0.012** |
| **Community Setting** | 153 |  |  |  |
| Not Urban |  | — | — |  |
| Urban |  | 0.03 | -0.25, 0.32 | 0.8 |
| **Years Practicing** | 153 | 0.06 | -0.05, 0.17 | 0.3 |
| **New Patients per Month** | 153 | 0.24 | 0.11, 0.37 | **<0.001** |
| **Tumor Board Frequency** | 153 | 0.17 | -0.05, 0.40 | 0.13 |
| **Enthusiasm about IDH inhibitors** | 153 | 0.08 | -0.04, 0.20 | 0.2 |
