# Supplementary material for: Contemporary practice patterns in IDH-mutant glioma management: a multidisciplinary multi-institutional survey
Source: J Neurooncol. 2026 Jun 8;178(2):54. doi: 10.1007/s11060-026-05630-3 (PMC13246546; doi:10.1007/s11060-026-05630-3)
Supplement: Supplementary file 9 — Supplementary Material 9 [file 11060_2026_5630_MOESM9_ESM.docx]

Supplementary Table 9: Univariable linear regression predicting enthusiasm for IDH inhibitors.

| Univariable Linear regression predicting IDHi Enthusiasm | | | | |
| --- | --- | --- | --- | --- |
| **Characteristic** | **N** | **Beta** | **95% CI** | **p-value** |
| **Practice Setting** | 153 |  |  |  |
| Not Academic |  | — | — |  |
| Academic |  | -0.26 | -0.71, 0.19 | 0.3 |
| **Specialty** | 153 |  |  |  |
| Neuro-Oncologist |  | — | — |  |
| Radiation Oncologist |  | -0.96 | -1.3, -0.66 | **<0.001** |
| Neurosurgeon |  | -0.21 | -0.71, 0.30 | 0.4 |
| Medical Oncologist |  | 0.57 | -0.09, 1.2 | 0.092 |
| **US Region** | 153 |  |  |  |
| West |  | — | — |  |
| Midwest |  | -0.39 | -0.86, 0.07 | 0.10 |
| Northeast |  | 0.06 | -0.36, 0.47 | 0.8 |
| South |  | -0.58 | -1.1, -0.07 | **0.025** |
| Outside US |  | 0.02 | -0.76, 0.81 | >0.9 |
| **Community Setting** | 153 |  |  |  |
| Not Urban |  | — | — |  |
| Urban |  | -0.32 | -0.70, 0.06 | 0.10 |
| **Years Practicing** | 153 | 0.12 | -0.03, 0.27 | 0.11 |
| **New Patients per Month** | 153 | 0.03 | -0.15, 0.22 | 0.7 |
| **Tumor Board Frequency** | 153 | -0.29 | -0.59, 0.01 | 0.056 |
| **Familiarity with IDH inhibitors** | 153 | 0.14 | -0.08, 0.36 | 0.2 |
